# HLA-C increases HIV-1 infectivity and is associated with gp120

**DOI:** 10.1186/1742-4690-5-68

**Published:** 2008-08-01

**Authors:** Andrea Matucci, Paola Rossolillo, Miriam Baroni, Antonio G Siccardi, Alberto Beretta, Donato Zipeto

**Affiliations:** 1 Laboratory of Molecular Biology, Department of Mother and Child, Biology and Genetics, Section of Biology and Genetics, University of Verona, Strada le Grazie 8, 37134, Verona, Italy; 2 University of Milan and DIBIT-San Raffaele Scientific Institute, Via Olgettina 58, 20132, Milan, Italy; 3 Infectious Diseases Department, IRCCS Ospedale San Raffaele, Via Stamira d'Ancona 20, 20127, Milan, Italy

## Abstract

**Background:**

A recently identified genetic polymorphism located in the 5' region of the HLA-C gene is associated with individual variations in HIV-1 viral load and with differences in HLA-C expression levels. HLA-C has the potential to restrict HIV-1 by presenting epitopes to cytotoxic T cells but it is also a potent inhibitor of NK cells. In addition, HLA-C molecules incorporated within the HIV-1 envelope have been shown to bind to the envelope glycoprotein gp120 and enhance viral infectivity. We investigated this last property in cell fusion assays where the expression of HLA-C was silenced by small interfering RNA sequences. Syncytia formation was analyzed by co-cultivating cell lines expressing HIV-1 gp120/gp41 from different laboratory and primary isolates with target cells expressing different HIV-1 co-receptors. Virus infectivity was analyzed using pseudoviruses. Molecular complexes generated during cell fusion (fusion complexes) were purified and analyzed for their HLA-C content.

**Results:**

HLA-C positive cells co-expressing HIV-1 gp120/gp41 fused more rapidly and produced larger syncytia than HLA-C negative cells. Transient transfection of gp120/gp41 from different primary isolates in HLA-C positive cells resulted in a significant cell fusion increase. Fusion efficiency was reduced in HLA-C silenced cells compared to non-silenced cells when co-cultivated with different target cell lines expressing HIV-1 co-receptors. Similarly, pseudoviruses produced from HLA-C silenced cells were significantly less infectious. HLA-C was co-purified with gp120 from cells before and after fusion and was associated with the fusion complex.

**Conclusion:**

Virionic HLA-C molecules associate to Env and increase the infectivity of both R5 and X4 viruses. Genetic polymorphisms associated to variations in HLA-C expression levels may therefore influence the individual viral set point not only by means of a regulation of the virus-specific immune response but also via a direct effect on the virus replicative capacity. These findings have implications for the understanding of the HIV-1 entry mechanism and of the role of Env conformational modifications induced by virion-associated host proteins.

## Background

A whole-genome association study of major genetic determinants for host control of HIV-1 has identified two polymorphisms that explain nearly 15% of the variation among individuals in viral load during the asymptomatic set-point period of infection. One of these polymorphisms is located in the 5' region of the HLA-C gene, 35 kb away from transcription initiation and has been reported to be associated with differences in HLA-C expression levels [1]. As a classical MHC class I gene, HLA-C has the potential to restrict HIV-1 by presenting epitopes to cytotoxic T cells (CTLs) [2,3], resulting in the destruction of infected cells. However, the potential ability of HLA-C to present epitopes to CTLs is severely limited by its poor expression at the cell surface (10-fold lower than either HLA-A or -B) [4] and its tendency to accumulate as free heavy chains or heavy chains associated with β_2_-microglobulin but free of peptides as a result of poor assembly [5]. HLA-C has also the least diversity of the three classical MHC class I loci. Accordingly, an analysis of the class I restricted CD8+T cell responses against HIV-1 revealed that variation in viral set-point and absolute T cell count is strongly associated with particular HLA-B, but not HLA-A or HLA-C allele expression [6]. In addition, HLA-Cw4/+ heterozygosity promotes rapid progression to AIDS illness, as does HLA-Cw4/Cw4 homozygosity [7]. Interestingly, the virus has evolved a strategy to selectively down-regulate HLA-A and -B but not HLA-C, via the regulatory protein Nef [8]. The immunity of HLA-C to Nef-mediated down modulation confers to the virus the capacity to escape NK cell attack since HLA-C is a dominant inhibitory ligand of NK cells [9]. Thus, the overall trade-off of high HLA-C expression might be favourable to the virus, and not to the host. The relative importance of CTLs and NK cells *in vivo *is still unclear and the interpretation of genetic studies showing association to viral set-point is particularly complex.

Like other MHC class I and II molecules, HLA-C is selectively incorporated into the HIV-1 envelope [10,11]. A study previously reported by our group [12] demonstrated that virion-associated HLA-C molecules have a profound influence on the infectivity of HIV-1. MHC class I negative cell lines were non permissive for the replication of primary HIV-1 isolates and only partially permissive for the replication of T cell line adapted viruses. Transfection of HLA-Cw4 into these cell lines restored their capacity to support viral replication. The increased infectivity of viruses grown in the presence of HLA-Cw4 was associated with changes in viral envelope protein conformation, which included an enhanced expression of epitopes not normally exposed upon CD4 binding.

Here we further investigate this phenomenon in a different experimental system where the expression of HLA-C was selectively silenced by small interfering RNA sequences (siRNA) and the infectivity-enhancement effect evaluated in fusion assays with cells expressing CCR5 and/or CXCR4 co-receptors. To overcome unknown effects of other viral gene products on viral infectivity, pseudotyped viruses expressing the same viral genome backbone, but different *env*, were used. The association of HLA-C with Env was tested using our previously reported technique for the detection of molecular complexes formed at the surface of cells during the fusion process (fusion complexes) [13].

## Results

### Effects of HLA-C on the HIV-driven fusion process

**Figure 1:**
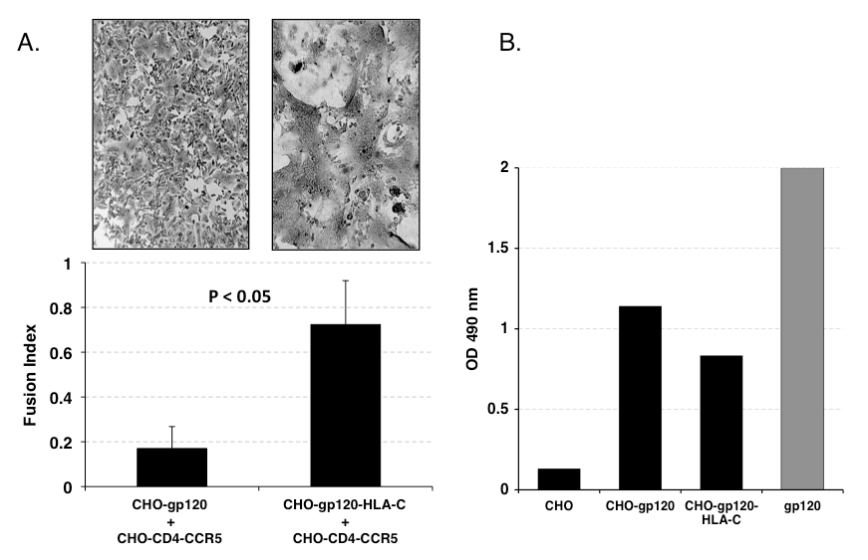
**Fusion efficiency of CHO cells expressing HLA-C and HIV-1 Env**. Panel A: Syncytia formation after co-cultivation of effector CHO cells expressing gp120/gp41 and HLA-C, or CHO cells expressing only gp120/gp41, with target CHO-CD4-CCR5 cells. The number and the extent of syncytia is significantly higher (p < 0.05) when effector cells express HLA-C. Panel B: ELISA analysis of Env expression. CHO, negative control; CHO-gp120, cells stably expressing the Env gene of the R5 tropic HIV-1 isolate 91US005; CHO-gp120-HLA-C: CHO-gp120 cells stably expressing HLA-Cw4; gp120: positive control, consisting of a mixture of five different gp120s. The higher fusion efficiency of CHO-gp120-HLA-C cells is not due to an increased level of Env expression, since they express 27% less gp120 than CHO-gp120 cells.

To assess the role of HLA-C in the fusion process we used a cell fusion assay between CHO cells expressing gp120/gp41, either alone or in combination with HLA-C and CHO cells expressing CD4-CCR5 (Table 1) [13]. When CHO-gp120-HLA-C cells were co-cultivated with CHO-CD4-CCR5 cells, a dramatic increase (p < 0.05) in the number and size of syncytia, as compared to those obtained with the same cells not expressing HLA-C, was observed (Fig. 1A). The increased fusion efficiency was not due to a higher expression level of gp120/gp41 in CHO-gp120-HLA-C cells, since they express on average 27% less gp120/gp41 than CHO-gp120/gp41 cells, when analyzed in ELISA using HIV-1 positive human sera (Fig. 1B).

**Table 1:** Summary of the HIV-1 envelopes tested in the different experimental models.

**Experimental model**	**Host cell** ** (HLA-C allele)**	**Env** ** (tropism/subtype)**
HLA-C siRNA silencing and cell fusion assays	HeLa (Cw12)	ADA (R5/B) LAI (X4/B) NDK (X4/D)

gp120/gp41 transient transfection and cell fusion assays	CHO-HLA-C (Cw4)	93MW965 (R5/C) 91US005 (R5/B) 92UG024 (X4/D) NDK (X4/D) J500 (X4/B)

Pseudovirus transductions	293T (Cw7)	pRHPA4259.7 (R5/B) 6535.3 (R5/B) NDK (X4/D) m7NDK (X4/D)

HLA-C silencing was conducted on human cells (HeLa-derived) physiologically expressing HLA-C and stably expressing Env of different strains (ADA, LAI, NDK).

Transient transfections experiments with plasmids encoding different Envs were conducted on non-human CHO cells stably expressing HLA-C to directly compare the effect of HLA-C in the absence of other human MHC class I molecules.

Pseudoviruses were produced in HLA-C silenced 293T cells since this human cell line is the election host for efficient and quantitative production of pseudotyped virus particle. The Envs tested belong to a standard reference panel (NIBSC EVA CFAR ARP2066) except NDK.

**Figure 2:**
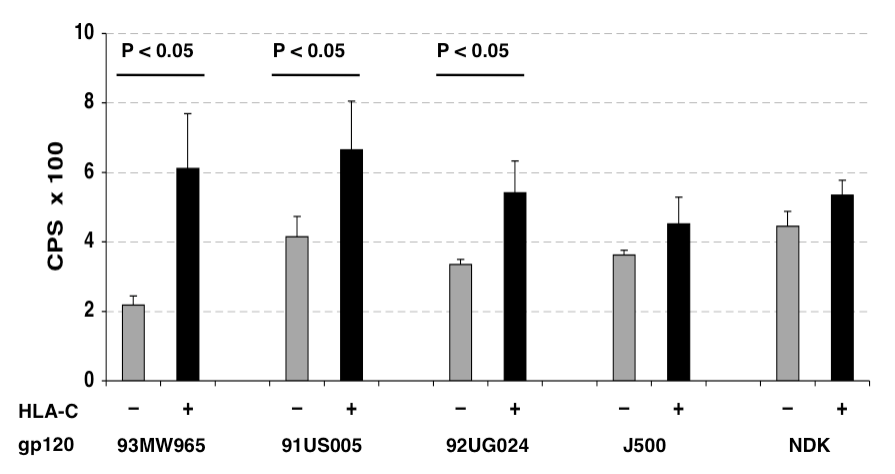
**Transient transfections of CHO cells expressing human HLA-C with different *env *sequences**. CHO (-, grey bars) and CHO-HLA-C (+, black bars) cells transiently transfected with plasmids encoding Tat, Rev and Env from different primary and laboratory HIV-1 isolates and co-cultivated for 6 hours with TZM-bl target cells. After Tat driven transactivation of firefly luciferase expression, fusion efficiency was quantified and expressed as counts per second (CPS). Each value represents the average of four replicates. The gp120/gp41 of primary isolates 93MW965 (R5), 91US005 (R5) and 92UG024 (X4) are HLA-C sensitive (p < 0.05) while isolates J500 (X4) and NDK (X4) are less sensitive to the presence of HLA-C (p not significant).

Similar results were obtained in a different cell fusion assay where CHO and CHO-HLA-C cells, transiently transfected with gp120/gp41 from different primary and laboratory HIV-1 isolates, were fused with TZM-bl cells and fusion quantified by luciferase transactivation. All gp120/gp41 tested (93MW965, 91US005, 92UG024) showed higher fusion efficiency when co-cultivated with TZM-bl cells if co-expressed with human HLA-C (Fig. 2). Only two X4-tropic isolates (J500 and NDK) failed to show a statistically significant fusion increase.

### HLA-C silencing of cells expressing gp120/gp41

HeLa cells constitutively express HLA-C and HLA-A and, at lower levels, HLA-B [14]. Various HeLa-derived cell lines, constitutively expressing HIV-1 Env, were silenced by HLA-C specific siRNAs (Table 1). The expression of gp120 in HeLa-ADA, -LAI and -NDK, as well as that of β_2_-microglobulin and GAPDH genes was not affected.

**Figure 3:**
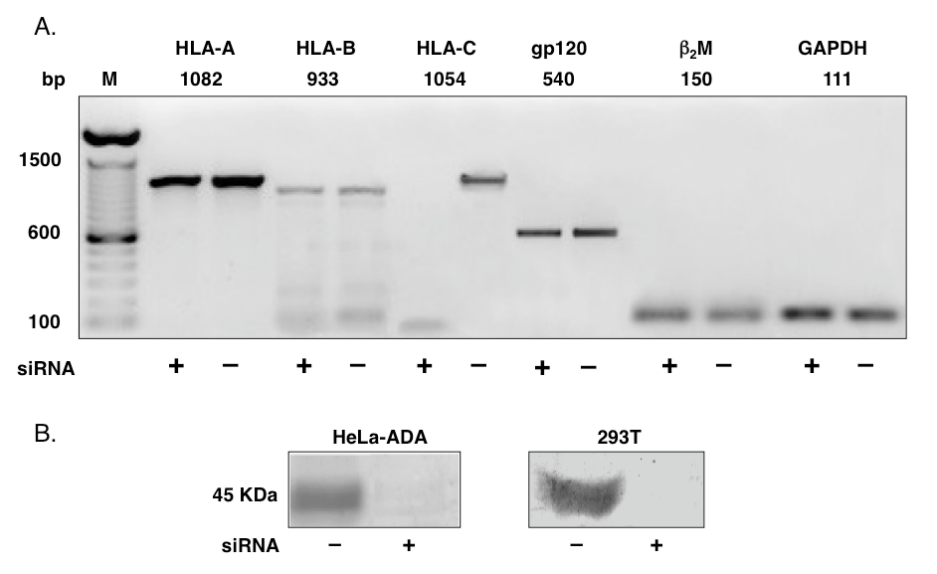
**Specific silencing of HLA-C in human cell lines**. Panel A: off-target effect analysis by RT-PCR in HLA-C silenced (+) and non-silenced (-) HeLa cells expressing HIV-1 gp120/gp41 (ADA). PCR was performed with primers specific for HLA (A, B, C), gp120, β_2_-microglobulin and GAPDH. M: molecular weight marker. No off-target effect due to HLA-C mRNA silencing is affecting the mRNA levels of the other MHC class I genes, as well as β_2_-microglobulin, HIV-1 gp120 or the housekeeping control gene GAPDH. Panel B: western-blot analysis of HLA-C protein expression. After 72 hours from siRNAs transfection, HLA-C is undetectable both in HeLa-ADA and in 293T cells.

**Figure 4:**
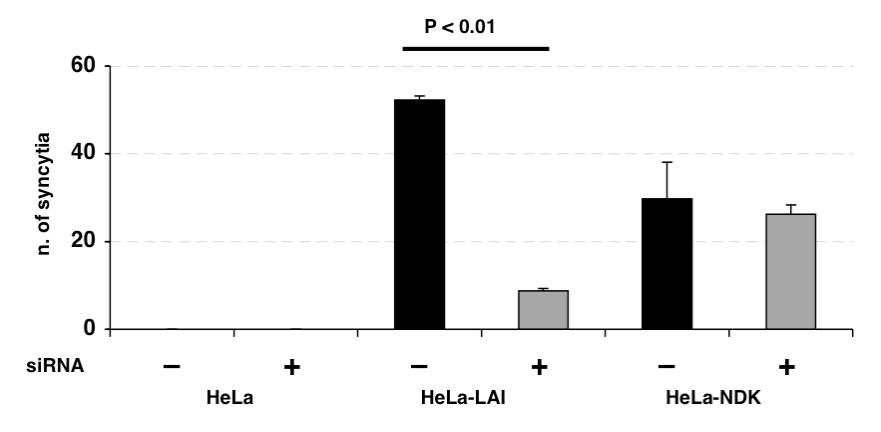
**Cell fusion of HLA-C silenced HeLa-Env cells with HeLa-P4.2 target cells**. Analysis of syncytia formation by co-cultivating HLA-C silenced (+) and non-silenced (-) HeLa-LAI and HeLa-NDK cells with target HeLa-P4.2 cells, expressing CD4 and CXCR4. The number of syncytia formed is lower (p < 0.01) using HLA-C silenced HeLa-LAI cells. Fusion efficiency of HeLa-NDK cells is not significantly affected by HLA-C silencing.

There was no unwanted off-target silencing of non HLA-C genes (Fig. 3A). The expression of HLA-C protein on HeLa-ADA and 293T cells was undetectable at 72 hours from siRNA transfection (Fig. 3B). Fusion efficiency, determined by counting the number of syncytia formed, was significantly lower (p < 0.01) when HLA-C silenced cells expressing HIV-1 gp120/gp41 of the LAI strain were co-cultivated with HeLa P4.2 cells as target cells (Fig. 4). Fusion efficiency of HeLa-NDK cells was less affected by HLA-C silencing, confirming that the NDK gp120/gp41 has a lower sensitivity to the presence of HLA-C [12]. When silencing was performed with siRNAs specific for HLA-C or with a pool of siRNAs silencing also HLA-A and -B, similar levels of reduction in fusion efficiency were observed.

### Syncytia formation using CCR5 or CXCR4 co-receptors

**Figure 5:**
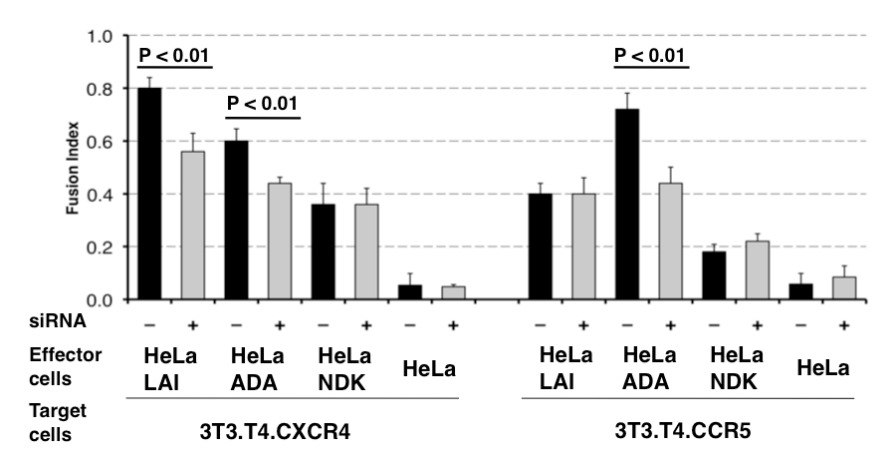
**Comparison of the fusion efficiency of HLA-C silenced HeLa-Env cells with 3T3.T4.CCR5 and 3T3.T4.CXCR4 cells**. HLA-C silenced (+, grey bars) and non-silenced (-, black bars) HeLa cells expressing gp120/gp41 of different HIV-1 isolates (ADA, LAI, NDK) co-cultivated with NIH 3T3.T4.CXCR4 and NIH 3T3.T4.CCR5 cells. Fusion efficiency of X4 tropic gp120 LAI is significantly lower (p < 0.01) in HLA-C silenced cells when fusing with CXCR4 target cells. Similarly, fusion efficiency of the R5 tropic gp120 ADA is lower (p < 0.01) in HLA-C silenced cells when fusing with CCR5 target cells. The fusion of ADA gp120 in HLA-C silenced cells with cells expressing CXCR4 is significantly (p < 0.01) less efficient, while that of LAI gp120 with cells expressing CCR5 is similar, irrespective of HLA-C silencing. The NDK gp120 is HLA-C insensitive, when using either the CXCR4 or the CCR5 co-receptor.

To test the role of HLA-C in the fusion process with cells expressing CCR5 or CXCR4 co-receptors, we measured the fusion index in co-cultures of HeLa-ADA and 3T3.T4.CCR5 cells or HeLa-LAI and 3T3.T4.CXCR4 cells with or without siRNA silencing of HLA-C. In both cultures, the fusion index was significantly lower (p < 0.01) in HLA-C-silenced cells than in the corresponding non-silenced controls (Fig. 5) showing that HLA-C increases the fusion efficiency of both CCR5 and CXCR4 tropic viruses.

3T3.T4.CXCR4 cells express 2–3 times more CXCR4 than HeLa-P4.2 and TZM-bl cells. Similarly, 3T3.T4.CCR5 cells express about 10 times more CCR5 as compared to TZM-bl cells (data not shown). We observed that these cells allowed the fusion with cells expressing Envs with a different co-receptor tropism, although at lower level. The use of the heterologous co-receptor, already evident [15] using pseudotyped viruses, is increased in fusion assays with Env-expressing cell lines, in particular for longer co-cultivation times. Under these experimental conditions, we investigated the role of HLA-C in modulating fusion efficiency in the presence of the heterologous co-receptor. We observed that the R5-tropic gp120/gp41 ADA was sensitive to HLA-C presence when fusing with 3T3.T4.CXCR4 cells whereas the X4-tropic LAI was not affected by HLA-C presence when fusing with 3T3.T4.CCR5 cells (Fig. 5). Also in these experiments, the NDK gp120/gp41 was found to fuse with the same efficiency with 3T3.T4.CXCR4 and, at lower levels, with 3T3.T4.CCR5 cells, when using HLA-C silenced or non-silenced HeLa-NDK cells (Fig. 5).

### Pseudovirus infection assay

**Figure 6:**
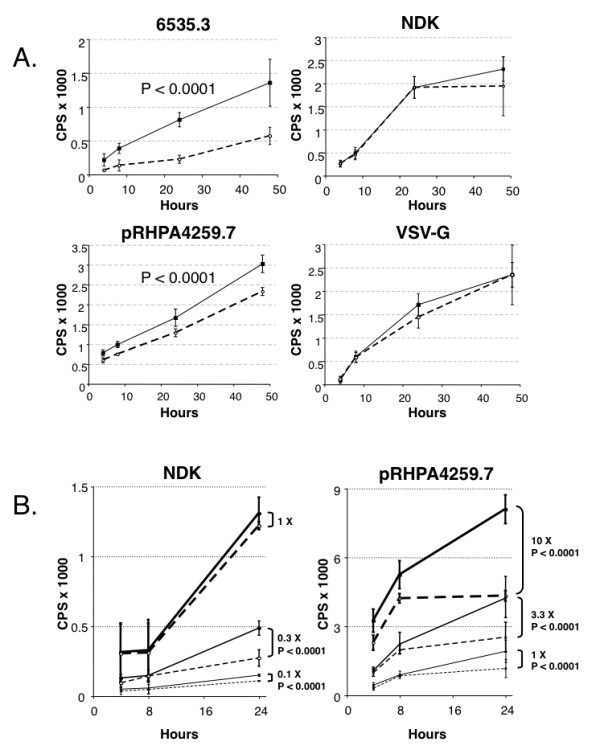
**Transduction efficiency of pseudoviruses produced in HLA-C silenced cells**. Panel A: luciferase reporter gene assay analysis after transduction with pseudoviruses expressing subtype B HIV-1 *env *(6535.3 and pRHPA4259.7) or subtype D HIV-1 *env *(NDK), produced in HLA-C silenced (dashed line, open circles) and non silenced (continuous line, close squares) 293T cells. Each point (expressed as counts per second, CPS) represents average and standard deviation of four replicates. HLA-C sensitive pseudoviruses 6535.3 and pRHPA4259.7 show a significant lower infectivity (p < 0.0001) when produced on HLA-C silenced cells. The NDK pseudovirus as well as a virus pseudotyped with the VSV-G envelope protein, do not show significant differences in infectivity when produced in HLA-C silenced or non silenced 293T cells. Panel B: analysis of the relation between pseudovirus infectious dose and HLA-C sensitivity. 1×, pseudovirus infectious titer giving a luciferase signal (expressed as counts per second, CPS) of 1000 at 16 hours post infection. When the HLA-C insensitive NDK pseudovirus was analyzed at lower infectious titers (0.3× and 0.1×), its infectivity was significantly increased by HLA-C. When the HLA-C sensitive pseudovirus pRHPA4259.7 was analyzed at higher infectious doses (3.3×, 10×), it remained sensitive to HLA-C presence.

Pseudoviruses produced on normal and HLA-C silenced 293T cells were quantified for p24 content and used in transduction assays (Table 1). Pseudoviruses bearing subtype B 6535.3 and pRHPA4259.7 HIV-1 *env *genes showed a statistically significant reduction in infectivity when produced in HLA-C silenced 293T cells. Conversely, no significant differences were observed with either NDK subtype D *env *gene or control virus pseudotyped with the VSV-G protein (Fig. 6A).

When the HLA-C insensitive NDK-pseudovirus was used at infectious doses that were 1/3 and 1/10 of the original inoculum, a significant infectivity difference between pseudoviruses produced in HLA-C silenced and non-silenced cells was noted. The HLA-C sensitive pseudovirus pRHPA4259.7 maintained its sensitivity to HLA-C also at lower m.o.i. (1/10 of the original inoculum, data not shown). When the m.o.i. of the pRHPA4259.7 pseudovirus was increased, the infectivity levels of pseudoviruses produced on normal and HLA-C silenced 293T cells was kept significantly different (Fig. 6B).

### HLA-C/gp120 association on cells before and after fusion

**Figure 7:**
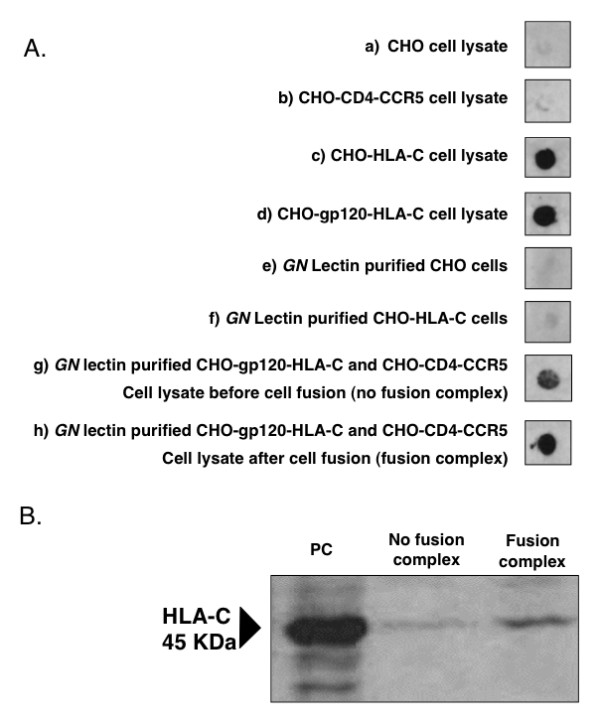
**Co-purification of fusion complexes containing HLA-C molecules**. Panel A: dot-blot analysis of purified fusion complexes for the presence of HLA-C. Lanes a, b, c and d: cell lysates before purification. Lanes e, f, g and h: cell lysates purified on *Galanthus nivalis *(GN) lectin columns. Panel B: western blot analysis to detect the presence of HLA-C in purified fusion complexes. Cells were treated with DTSSP, which fixes only proteins present on the cell membrane, and lysates purified on GN lectin columns. PC: positive control (HeLa cells expressing HLA-C); the arrow indicates HLA-C.

In the previous study we provided evidence of a specific association between virionic HLA-C molecules and gp120 by co-immunoprecipitating the two molecules with the HLA-C-specific monoclonal antibody L31 and a gp120-specific antibody [12]. In this work we looked for additional evidence of HLA-C-gp120 association occurring on cells taken after fusion using a previously described method that allows the isolation of CD4-CCR5-gp120/gp41 fusion complexes after fixation with paraformaldehyde or DTSSP and purification with *Galanthus nivalis *(*GN*) lectin, which specifically binds to gp120 [13]. The presence of HLA-C molecules within the fusion complexes could be tested by dot blot with the antibody L31 which also recognizes the denatured protein [16]. Fig. 7 panel A shows a dot-blot with antibody L31 of total cell lysates or proteins eluted from *GN *lectin columns. L31-reactive molecules were detected in total cell lysates of CHO-HLA-C (lane c) and CHO-gp120-HLA-C cells (lane d) but not in the HLA-C negative CHO cell line (lane a) and the CHO-CD4-CCR5 fusion partner (lane b). The eluate of *GN *lectin columns loaded with a mixed extract of CHO-gp120-HLA-C and CHO-CD4-CCR5 cells which had been fixed before fusion, displayed a significant amount of L31 reactive molecules (lane g), showing that a specific association between HLA-C and gp120 occurred in cells co-expressing the two molecules, as previously described in LAI-infected 221-Cw4 cells [12]. When the same cells were allowed to fuse before being fixed, the eluate of *GN *lectin purified cell extract displayed an increased amount of L31-reactive molecules (lane h) indicating that during the process of cell fusion additional HLA-C molecules are recruited within the fusion complexes. The lack of L31-reactive molecules in the eluate of *GN *lectin purified CHO-HLA-C cells (lane f) demonstrates that in this experimental setting HLA-C molecules are purified via their specific binding to gp120.

To gain further evidence of the association between HLA-C and gp120, the same protein samples, after fixation with DTSSP and purification on *GN *lectin columns, were chemically reduced, separated on SDS-PAGE and blotted with L31 antibody which revealed a 45 kDa band corresponding to the HLA-C heavy chain. Also in this experiment a relatively higher amount of HLA-C was co-purified from cells which were allowed to fuse before fixation (fusion complex), as compared to non-fused cells (no fusion complex) (Fig. 7B). These results provide further evidence that HLA-C is associated to gp120 on the cell membrane and suggest that additional HLA-C is recruited within the fusion complex during cell fusion.

### Sequence analysis of HLA-insensitive Envs

The sequence of the *env *gene of the HLA-C insensitive primary isolate J500 (clade B) was determined. When this was compared to the sequence of the other HLA-C insensitive isolate NDK (clade D), and to the sequences of the HLA-C sensitive Envs tested (93MW965, 91US005, 92UG024, ADA, LAI, 6535.3 and pRHPA4259.7), three identical aminoacid substitutions (N297K, N298Y and I318T, relative to the LAI *env *sequence) were identified in the V3 loop. *Env *sequence analysis of the Los Alamos HIV Reference Database showed that the I318T mutation is relatively uncommon, occurring in 92 out of the 1603 *env *sequences available (5.7%). Mutations N297K and N298Y are extremely rare, occurring only in 2 isolates reported in the database. In addition, the combination of these 3 mutations was found only in a single *env *sequence (isolate D.TZ.87.87TZ4622). Position 297 is associated with a potential N-glycosilation site [17].

## Discussion

This work demonstrates that virion-associated HLA-C molecules, when present on cells expressing gp120/gp41, significantly enhance fusion efficiency and pseudovirus transduction. Our conclusions are supported by the following findings: a) CHO cells co-expressing HIV-1 gp120/gp41 and human HLA-C fuse more rapidly and produce larger syncytia than the original CHO-gp120/gp41 cells from which they are derived; b) transient transfection of gp120/gp41 from different primary isolates in CHO cells co-expressing HLA-C results in a significant increase in fusion; c) silencing of HLA-C in human cell lines expressing HIV-1 gp120/gp41 of R5 and X4 tropic strains, significantly suppresses fusion, d) pseudoviruses produced in HLA-C silenced 293T cells display a significant reduction of infectivity; e) the fusion enhancement property of HLA-C is specific for HIV-1 Env, since a virus pseudotyped with the G envelope protein of VSV is not influenced by the presence of HLA-C.

The effect of HLA-C on fusion was observed with both exogenous HLA-C transfected into CHO cells and endogenous HLA-C after its silencing with siRNA in human cells.

Some of the data point to the existence of HLA-C "insensitive" or "less-sensitive" variants since the fusogenic capacity of gp120/gp41 from two isolates, NDK and J500, was not different in HLA-C-silenced and non-silenced cells. However, we observed that HLA-C insensitivity is not an absolute feature, since there was a small difference, although not statistically significant, in the fusion efficiency of NDK and J500 in silenced and non-silenced cells. In addition, a relationship between the infectious dose and the HLA-C sensitivity of pseudoviruses was observed since when infections were performed at low infectivity ratios, the HLA-C insensitive NDK pseudovirus became HLA-C sensitive. Conversely, when high titers of an HLA-C sensitive pseudovirus were used, its infectivity remained dependant on the presence of HLA-C. The relative insensitivity of NDK to the presence of HLA-C could contribute to its reported higher cytopathicity and infectivity [18] and could be the result of a variable infectivity degree of Env [12] or of a lower level of incorporation of HLA-C [10].

The comparison of the *env *sequences of the two unrelated, HLA-C insensitive gp120/gp41s identified, NDK (clade D) and J500 (clade B), with the sequences of the HLA-C sensitive Envs, revealed 3 identical aminoacid substitutions in the V3 loop, which were absent in all other HLA-C sensitive Envs analyzed. This would suggest an involvement of these mutations in the V3 loop in the acquisition of the HLA-C insensitive phenotype. We analyzed an NDK-derived Env mutant, NDKm7 [19], in which the KY mutations in position 297–298 reverted to NN. Virus particles pseudotyped with the NDKm7 *env *remained HLA-C insensitive as the original NDK *env *(data not shown), thus excluding the involvement of these mutations in reducing sensitivity to HLA-C presence. It is possible that other mutations, or their combinations, might directly affect the sensitivity to HLA-C by changing the pattern of interaction between HLA-C and gp120, as reported by other authors who studied mutations related to the acquisition of a CD4-independent tropism within gp120 [19,20].

The data reported in this study confirm the physical association between HIV-1 gp120/gp41 and HLA-C, that was originally observed in experiments in which HLA-C and gp120 were co-immunoprecipitated from HIV-1 infected cells [12]. HLA-C molecules could be co-purified and detected in fusion complexes in association with gp120/gp41, CD4 and the co-receptor. Such an association may induce conformational changes of gp120 favouring the exposure of cryptic functional epitopes [12]. It has also been recently reported that viral particles carry more HLA molecules than gp120/gp41 trimers [21]. The association between a gp120/gp41 trimer and multiple HLA-C molecules might reduce gp120 shedding, thus keeping more functional the trimeric gp120/gp41 complexes on the viral envelope and resulting in increased fusion efficiency.

The increase in fusion and viral infectivity was observed using CHO cells transfected with HLA-Cw4, as well as HeLa cells which express constitutively HLA-Cw12 and pseudoviruses originating from 293T cells which express HLA-Cw7 (Table 1). Similar results were obtained with the HLA-Cw3 allele (L. Lopalco, DIBIT-San Raffaele, Milan, personal communication). Altogether, the Cw3, Cw4, Cw7, and Cw12 serological alleles include members of both groups of the known HLA-C dimorphism [22] and account for almost 80% of all the common HLA-C serotypes. Due to the more limited polymorphism of HLA-C as compared to HLA-A and -B, this limited panel is inclusive enough to allow us to sample all the HLA-C-distinctive substitutions and most of the common allelic variations. Remarkably, most of these cluster around the binding groove, but the co-immunoprecipitation of env with HLA-C [12] was observed by immunoprecipitating the complex with antibody L31, that binds on the alpha 1 domain alpha helix, e. g. in proximity to the sites at which essentially all the polymorphic HLA-C positions cluster. This suggests that HLA-C polymorphism is unlikely to influence this association, and that the residues important for co-immunoprecipitation reside within the relatively invariant HLA-C backbone. In line with this finding, we have observed the infectivity-enhancement effect with all the alleles tested so far, suggesting that most HLA-C alleles bind Env. We cannot however exclude the possibility that some HLA-C allelic variants may be more efficient than others in binding Env and enhancing viral infectivity.

An implication of these findings is that HLA-C may be selectively involved in protective immunity. A protective effect was observed in HIV serodiscordant couples with unmatched HLA-C alleles [23] and anti-HLA antibodies are frequent in exposed, but seronegative subjects [24,25]. It has also been reported that MHC class I concordance is associated with an increased risk of mother to child HIV-1 transmission [26,27]. Since early studies in primates were suggestive of anti-MHC antibodies being protective [28], the possibility of using HLA molecules for a HIV-1 vaccine has long been debated [29,30]. Our data point to an association between HLA-C and Env in mature virions which may induce the expression of critical conformational epitopes [12]. Since the few Env that showed lower sensitivity to HLA-C are X4 tropic, the inclusion of HLA-C in new immunogenic formulations may help eliciting broadly neutralizing antibodies that would be important for the *in vivo *host control of R5 tropic strains of HIV-1.

## Conclusion

HLA-C influences viral replication by at least three distinct and opposite mechanisms: induction of cytotoxic T cells (suppression), inhibition of NK cells (enhancement) and enhancement of virus infectivity. This last effect is associated to a specific association of virionic HLA-C molecules to Env. The immunity of HLA-C to the Nef-induced down-regulation confers to the virus not only the capacity to escape NK cells control but also a higher replicative capacity suggesting that high HLA-C expression is advantageous to the virus and not the host.

## Methods

### Antibodies

W6/32 is a mouse monoclonal antibody specific for HLA-A, -B and -C trimeric complex [31]. The L31 monoclonal antibody is specific for the α domain of HLA-C heavy chain [32-34], not associated to β_2_-microglobulin. Anti-gp120 human sera from HIV-positive patients were kindly provided by Dr. Lucia Lopalco, DIBIT-HSR, Milan, Italy. IgG were purified using Protein G Sepharose 4 Fast Flow (GE Healthcare Lifescience, Chalfont St. Giles, UK) following manufacturer's instructions.

### Cells

HeLa (HLA-Cw12, [35]) and HEK-293T (HLA-Cw07, [35]) cells were obtained from the American Type Culture Collection (ATCC).

HeLa-derived effector cell lines expressing the HIV-1 *env *gene of strains ADA, LAI [36] and NDK [37] and the indicator target cell line HeLa P4.2 [38] were kindly provided by Dr. Mark Alizon and Dr. Uriel Hazan, Institut Cochin, Paris, France.

NIH 3T3 cells expressing the HIV-1 receptor CD4 and the chemokine receptor CCR5 (3T3.T4.CCR5) or CXCR4 (3T3.T4.CXCR4) were obtained from the NIH AIDS Research & Reference Reagent Program, division of AIDS, NIAID, Dr. Dan R. Littman [15].

The TZM-bl cell line [39] was from the EU programme EVA/MRC, CFAR NIBSC, UK. This cell line expresses CD4, CCR5 and CXCR4 and contains HIV-1 LTR-driven *E. coli *β-galactosidase and firefly luciferase reporter cassette that are activated by HIV-1 Tat expression.

CHO and CHO-gp120/gp41 [13] cells were stably transfected with the vector pZeoSV2(+) (Invitrogen, Carlsbad, CA, USA) bearing the HLA-Cw4 gene, and the cell lines obtained were named CHO-HLA-C and CHO-gp120-HLA-C, respectively.

CHO and CHO-HLA-C cell lines were transiently transfected with HIV-1 *env *genes from primary and laboratory isolates NDK, J500 (a primary X4 tropic isolate [40]), 92UG024, 93MW965 and 91US005 [41] cloned in the expression vector pCDNA3.1 (Invitrogen, Carlsbad, CA, USA).

### RNA silencing of HLA-C

The HLA-C mRNA [GenBank: NM_002117] target sites for siRNA were determined by using the Dharmacon siGENOME software and synthesized by Dharmacon (Lafayette, CO, USA). The siRNAs targeted different regions of the HLA-C mRNA.

In particular, siRNAs J-017513-06 (5'P-UAAUCCAUCAACGCUUCAUUU-3') and J-017513-08 (5'P-UUUGGAAGGUUCUCAGGUCUU-3') were found to be specific for HLA-C silencing, while siRNAs J-017513-05 (5'P-AUAGCGGUGACCACAGCUCUU-3') and J-017513-07 (5'P-ACUUCUAGGAAUUGACUUAUU-3') also silenced HLA-A and -B mRNAs.

HeLa cells expressing *env *genes were transfected with 100 nmol/well of siRNA following manufacturer's instructions, using DharmaFECT 1 reagent (Dharmacon, Lafayette, CO, USA). The silencing of HLA-C protein expression was verified by Western blot after 72 hours.

The absence of off-target effects was verified both by RT-PCR of HLA-A, -B, -C, β_2_-microglobulin, HIV-1 *env *and GAPDH, and by ELISA analysis of gp120/gp41 expression using HIV-1 positive human sera.

### TZM-bl reporter gene assays

The fusion process between gp120/gp41 effector cells (HeLa-ADA, HeLa-LAI, HeLa-NDK) and TZM-bl cells was assessed by measuring luciferase activity and by X-gal cell staining.

TZM-bl cells (50.000 per well) were plated in 96 microtiter wells (Corning, NY, USA) to an equivalent number of effectors cells for 3 to 6 hours at 37°C. The luciferase activity resulting from fusion and transactivation was analyzed using the Brite Lite reagent following manufacturer's instructions and quantified by using a Victor 3 apparatus (Perkin Elmer, Waltham, MA, USA). All the assays were performed in triplicate.

*In situ *staining of fusing cells for β-galactosidase gene activation was performed in a 24-well plate format (Corning Life Sciences, Lowell, MA, USA) as reported [42]. Blue-stained syncytia were photographed using a Nikon Eclipse 80 *i *microscope, counted and fusion efficiency determined by calculating the fusion index [43].

### Cell fusion assays

NIH 3T3.T4.CCR5 and 3T3.T4.CXCR4 were stained with the fluorescent lipophilic dye Vybrant DiI (Invitrogen, Carlsbad, CA, USA) following manufacturer's instructions. Cells were plated at 400,000 per well on a six-well plate (Corning Life Sciences, Lowell, MA, USA) and, 72 hours post siRNA transfection, co-cultivated at 1:1 ratio with HLA-C silenced and non-silenced HeLa-gp120/gp41 cells labeled with the fluorescent lipophilic dye Vybrant DiO (Invitrogen). After 6 hours, syncytia formation was analyzed using a fluorescence microscope (Nikon Eclipse *80i*) for green and red fluorescence and the double positive yellow syncytia counted [44,45].

### gp120 ELISA detection assay

Ninety-six well plates (Nunc, Roskilde, Denmark) were coated with 50 μl/well of a solution of 2 μg/ml of the D7324 gp120 antibody (Aalto Bioreagents, Dublin, Ireland), and a 3 mg/ml solution of total protein from cell lysate samples was added as described [13]. Positive controls consisted of a 100 ng/ml pool of 5 different gp120s obtained from EVA/MRC Centralised Facility for AIDS Reagents, NIBSC, UK (CN54, IIIB, MN, SF2 and W61D). Plates were washed and incubated with 1:200 diluted purified human IgG from sera of HIV-1 positive patients (25 mg/ml), washed and incubated with 1:500 diluted goat anti-human horseradish peroxidase conjugate (BioRad). Optical signal was developed with SigmaFast OPD solution (Sigma, St. Louis, MO, USA).

### RT-PCR

Total RNA was extracted from 24 hours silenced and non-silenced cultured cells using the RNeasy Plus mini kit (Qiagen, Germantown, MD, USA) and treated with RNase-free DNase I (Sigma). Reverse transcription (RT) of 1 μg of total RNA was performed using the Quantitect Reverse Transcription kit (Qiagen) and random primers. For PCR amplification of HLA-A, -B and -C, primers and conditions were used as previously reported [14]. Primers used to amplify HIV-1 *env *gene were: 5'-GGGCCACACATGCCTGTGTA-3' forward and 5'-CTAATTCCATGTGTACATTGTACTGTG-3' reverse; for β_2_-microglobulin amplification 5'-GATGAGTATGCCTGCCGTGTG-3' forward and 5'-CAATCCAAATGCGGCATCT-3' reverse; for glyceraldehyde-3-phosphate dehydrogenase (GAPDH) amplification 5'-GCATCCTGGGCTACACTGA-3' forward and 5'-TGACAAAGTGGTCGTTGAGG-3' reverse. PCR was performed for 32 cycles at 94°C, 60°C and 72°C for 1 min in each step. PCR products were analyzed on a 1% agarose gel and stained with Sybr Safe (Molecular Probes, Eugene, OR, USA). Images were acquired with an AutoChemi System apparatus (UVP, Cambridge, UK). Controls for genomic DNA contaminations consisted in RT reactions in which the polymerase was omitted.

### Western blot analysis

Seventy-two hours after HLA-C siRNA transfection, cells were lysed, the total protein content of supernantant was measured using a colorimetric assay (DC protein assay, BioRad, Hercules, CA, USA) and used for western blot analysis.

Equal amounts (30 μg/lane) of cell lysates were separated on 3 to 8% NuPAGE Tris-acetate acrylamide gels (Invitrogen, Carlsbad, CA, USA) and transferred onto polyvinylidene difluoride membranes (GE Healthcare Lifescience, Chalfont St. Giles, UK). Membranes were blocked in a Tris-buffered saline solution containing 5% non-fat dry milk and incubated with the L31 monoclonal antibody (1:200 dilution). Anti-mouse horseradish peroxidase-conjugated antibody (Dako, Carpinteria, CA, USA) was used as secondary antibody at 1:2,000 dilution and immunoreactive bands were visualized with the Opti-4CN detection kit (BioRad, Hercules, CA, USA).

### FACS analysis

Cells were analyzed by immunofluorescent staining and cytofluorimetry on a FACScanto apparatus (Becton Dickinson, San Jose, CA, USA). After incubating 500,000 cells with the primary anti-HLA monoclonal antibodies W6/32 or L31, these were washed and incubated with a 1:200 dilution of the goat-anti mouse IgG-FITC secondary antibody (Becton Dickinson). The analysis was conducted using the FACSDiva software (Becton Dickinson). For L31 epitope unmasking through β_2_-microglobulin stripping, cells were pre-treated with acidified medium as described [46] and immediately analysed.

### Infectivity of pseudoviruses produced on HLA-C silenced cells

HLA-C mRNA was silenced in 293T cells as previously described for HeLa cells. After 24 hours, silenced and non-silenced 293T cells were co-transfected with backbone (pSGΔenv) and *env *plasmids (subtype B isolates 6535.3 and pRHPA4259.7, subtype D isolate NDK, and Vescicular Stomatitis Virus (VSV) envelope protein G), as described [47]. Pseudoviruses were collected after 48 hours and quantified for p24 content using a standard ELISA Kit following manufacturer's instructions (Innotest-HIV antigen mAb, Innogenetics, Gent, Belgium). Both normal and HLA-C silenced pseudoviruses were used at a p24 concentration of 150 pg/ml. Infections of TZM-bl cells were done in quadruplicate and luminescence measured after 4, 8, 24 and 48 hours of incubation using a Victor 3 luminometer (Perkin Elmer) as previously described.

### Fusion complex analysis

Fusion complexes were fixed with paraformaldehyde, purified and analyzed as described [13]. Briefly, CHO-gp120-HLA-C and CHO-CD4-CCR5 cells were co-cultivated 4 hours at 37°C, fixed and lysed. Cell lysates were passed over a snowdrop *Galanthus nivalis *lectin column and eluted in 1 M methyl α-D-mannopyranoside (Sigma). Fusing cells were also fixed with DTSSP (Pierce Biotechnology, Rockford, IL, USA), following manufacturer's instructions. Fusion complexes were purified and dissociated using 5% β-mercaptoethanol in SDS-PAGE sample buffer. Effector and target cells were also separately fixed prior purification. Paraformaldehyde fixed fusion complexes were analysed for HLA-C co-purification by dot-blot and DTSSP fixed complexes by Western blot with HLA-C specific mAb L31.

### Statistical analysis

Data were analyzed by ANOVA and unpaired Student's t-test with Welch's correction, using the software GraphPad Prism 4.0c (GraphPad Software, Inc., CA, USA).

### Sequence analysis

HIV-1 Env sequences (NDK [GenBank: A34828], LAI [GenBank: AF004394]; ADA [GenBank: AY426119]; 92UG024 [GenBank: U43386]; 93MW965 [GenBank: U08455]: 91US005 [GenBank: U27434]) were aligned and compared using CLC Sequence Viewer 4.6.2, developed by CLC bio A/S  for Apple Mac OSX.

## Competing interests

The authors declare that they have no competing interests.

## Authors' contributions

AM carried out siRNA silencing, RT-PCR, cell transfections, ELISA, Western blot and FACS analysis, cellular fusions and pseudovirus infections experiments. PR carried out sequencing, pseudovirus preparation and titration and fusion complexes preparation and analysis. MB isolated and cloned the HLA-C insensitive *env *sequence from the J500 primary isolate. AGS participated in the design and coordination of the study and drafted the manuscript. AB participated to study design, data analysis and gave a significant contribution in drafting and revising the manuscript. DZ produced Env-coding plasmids and stably transfected cell lines, did fusion complexes preparation and analysis, conceived the study and carried out its design, and, as corresponding author, carried out the drafting of the manuscript. All authors read and approved the final manuscript.
